# A new metabolomic assay to examine inflammation and redox pathways following LPS challenge

**DOI:** 10.1186/1476-9255-9-37

**Published:** 2012-10-04

**Authors:** Jung H Suh, Robert Y Kim, Daniel S Lee

**Affiliations:** 1 Nutrition and Metabolism Center, Children’s Hospital Oakland Research Institute, Oakland, CA, USA

**Keywords:** Macrophage, Lipopolysaccharide, Sub-clinical inflammation, Arginine metabolism, Redox regulation, Biomarker

## Abstract

**Background:**

Shifts in intracellular arginine (Arg) and sulfur amino acid (SAA) redox metabolism modulate macrophage activation, polarization and phenotype. Despite their importance in inflammation and redox regulatory pathways, comprehensive analysis of these metabolic networks was not previously possible with existing analytical methods.

**Methods:**

The Arg/thiol redox LC-MS/MS metabolomics assay permits simultaneous assessment of amino acids and derivative products generated from Arg and SAA metabolism. Using this assay, LPS-induced changes in macrophage amino acid metabolism were monitored to identify pathway shifts during activation and their linkage to cellular redox regulation.

**Results:**

Metabolite concentrations most significantly changed after treatment of a macrophage-like cell line (RAW) with LPS for 24 hrs were citrulline (Cit) (48-fold increase), ornithine (Orn) (8.5-fold increase), arginine (Arg) (66% decrease), and aspartic acid (Asp) (73% decrease). The ratio Cit + Orn/Arg + Asp (CO/AA) was more sensitive to LPS stimulation than other amino acid ratios commonly used to measure LPS-dependent inflammation (e.g., SAM/SAH, GSH/GSSG) and total media NOx. The CO/AA ratio was also the first ratio to change significantly after LPS treatment (4 hrs). Changes in the overall metabolomic profile over time indicated that metabolic pathways shifted from Arg catabolism to thiol oxidation.

**Conclusions:**

Simultaneous quantification of Arg and SAA metabolic pathway shifts following LPS challenge of macrophage indicate that, in this system, the Arg-Citrulline/NO cycle and arginase pathways are the amino acid metabolic pathways most sensitive to LPS-challenge. The cellular (Cit + Orn)/(Arg + Asp) ratio, which summarizes this pathway, was more responsive to lower concentrations of LPS and responded earlier than other metabolic biomarkers of macrophage activation including GSH redox. It is suggested that the CO/AA ratio is a redox- independent early biomarker of macrophage activation. The ability to measure both the CO/AA and GSH-redox ratios simultaneously permits quantification of the relative effects of LPS challenge on macrophage inflammation and oxidative stress pathways. The use of this assay in humans is discussed, as are clinical implications.

## Background

While a direct causal link between inflammation/oxidative stress and disease has not been proven, heightened systemic oxidative and inflammatory stress is strongly associated with the transition from health to disease [1-3]. For example, prospective epidemiological studies indicate that increases in oxidative stress and inflammation are associated with elevated risk of cognitive decline, cardiovascular disease, and cancer [4-8].

**Figure 1:**
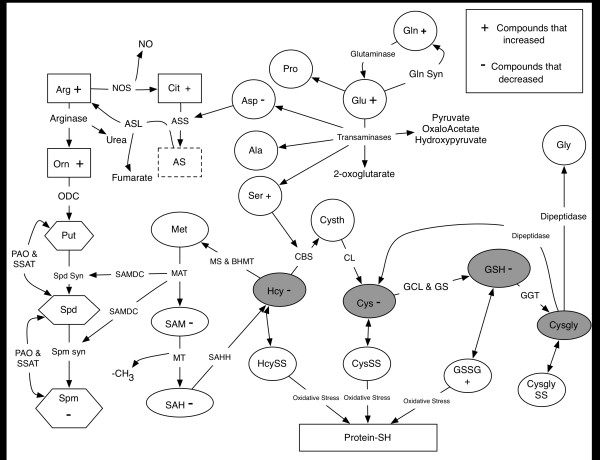
**Metabolites and their metabolic pathway networks measured by the Arg/Thiol Redox Metabolomics Assay.** The assay covers nearly all Arg metabolic pathways (rectangle), including the arginase, Cit-NO cycle, and polaymine pathways (hexagon). Also shown are sulfur amino acid derived sulfhydryl containing redox metabolites (grey ovals), and names of enzymes involved in the synthesis pathways. Argininosuccinate (dotted rectangle box) was not measured. Some major amino acids (circles) were measured but are not shown in this figure (See Table 1 for a complete list of analytes quantified). Compounds that were significantly decreased or increased after 24 hrs of treatment with LPS (1000 ng/ml) are highlighted with - or +, respectively. Abbreviations: Alanine – Ala; Arginine – Arg; Asparagine – Asn; Aspartate – Asp; Argninosuccinate Lyase - ASL; Argininosuccinate Synthetase – ASS; Betaine Hydroxymethyl Transferase - BHMT; Citrulline – Cit; Cysteine – Cys; Cystathionine Beta-synthase - CBS; Cystathionine Lyase - CL; Î³-Glutamyl Transpeptidase – GGT; Î³-Glutamylcysteine Ligase – GCL; Glutathione – GSH; Glutathione disulfide – GSSG; Glutathione synthase - GS ; Glutamate – Glu; Glutamine – Gln; Glutamine Synthase - Gln Syn; Glycine – Gly; Histidine – His; Homocysteine – Hcy; Methionine – Met; Methionine Adenosyl Transferase – MAT; Methyl Transferase - MT; Methionine Synthase - MS; Nitric Oxide Synthase - NOS; Ornithine – Orn; Ornithine Decarboxylase - ODC; Proline - Pro; Putrescine – Put; Polyamine Oxidase - PAO; S-adenosylmethionine – SAM; S-Adenosylmehtionine Decarboxylase - SAMDC; S-adenosylhomocysteine – SAH; S-Adenosylhomocysteine hydrolyase - SAHH; Serine – Ser; Spermidine – Spd; Spermidine Synthase - Spd Syn; Spermine synthase - Spm Syn; Spermine – Spm; Spermidine/Spermine N1 acetyltransferase - SSAT.

Non-proteinogenic metabolism of amino acids is a central part of the inflammation and redox environment. Arginine (Arg) and sulfur amino acid (SAA) metabolism play critical roles in regulating the physiological response to injury and oxidative stress [9-12]. These pathways are illustrated in Figure 1 and briefly described below.

Arginine (Arg) is a conditionally essential amino acid whose nutritional requirement is increased during inflammation [9-11]. During inflammation, Arg is catabolized primarily to citrulline (Cit) (Figure 1: rectangle) and ornithine (Orn) (Figure 1: rectangle) to generate NO, urea and the polyamines putrescine (Put), spermidine (Spd), and spermine (Spm) (Figure 1: hexagons) [9-11]. The products of Arg catabolism directly modulate inflammation by controlling endothelial function, leukocyte recruitment and activation, innate immunity, and extracellular matrix remodeling [9-11].

Metabolism of sulfur amino acids (SAA) occurs through four interconnected metabolic pathways - transmethylation, transsulfuration, GSH synthesis, and the γ-glutamyl-cycle (Figure 1: ovals) [13,14]. As shown, each of these cycles produce one sulfhydryl (−SH) containing compound. These are, respectively, homocysteine (Hcy), cysteine (Cys), glutathione (GSH) and cysteinylglycine (Cysgly) (Figure 1: grey ovals). The homeostatic balance of these compounds is important for cellular redox regulation [15,16]. Excess accumulation of Hcy and Cys is pro-inflammatory in cells [17-20] and is also associated with increased cardiovascular disease risk [12,21-23]. GSH is the principal thiol antioxidant, and a key regulator of cellular protein thiol redox states [15,16]. In addition to its direct antioxidant function, the synthesis of GSH from its rate-limiting substrate Cys minimizes the toxicity of excess Cys accumulation and acts as a cellular Cys reserve [15,16]. In fact, the release of Cys on GSH breakdown is an important means of inter-organ Cys trafficking [24]. GSH is only catabolized by γ-glutamyl transpeptidase (GGT), which generates Cysgly dipeptide [24]. Microsomal dipeptidase enzymes hydrolyze Cysgly to yield Cys. Because Cysgly does not accumulate in cells, it does not contribute significantly to cellular redox regulation directly. GGT expression is increased in conditions of inflammation and oxidative stress and acts as an important part in the cellular redox regulation [25-27].

The Arg and SAA metabolic pathways are also impacted by metabolism of amino acids that are not directly a part of those pathways. For example, on Arg deficiency, amino acids such as aspartate, glutamate and proline (Figure 1: circles) can be utilized to regenerate Arg [28,29]. Cystathionine (Cysth), a precursor of Cys, is synthesized from homocysteine and serine [13,14]. Gly and Glu are also required for GSH formation. Thus, a comprehensive analysis of amino acids, in addition to directly focusing on metabolites in the Arg and SAA pathways, may be useful in identifying factors that contribute to Arg and SAA pathway shifts on inflammation and oxidative stress.

While several sensitive analytical techniques are available that quantify GSH redox states and secondary Arg metabolic intermediates [30-35], the method utilized here is the first that permits simultaneous monitoring of all amino acids and the major secondary metabolites in both Arg and SAA metabolism.

Our Arg/thiol redox metabolomics assay combines an isopropylchloroformate derivatization method previously developed for analyzing 20 major amino acids [36] with modifications that enable detection of reduced and oxidized thiols with minimal ex-vivo oxidation [37]. The assay targets the major amino acids and secondary metabolic products of Arg and SAA shown in Figure 1, a total of 34 metabolites. This report presents results of experiments using RAW murine macrophage-like cells to test the sensitivity of this assay in detecting metabolic shifts in both oxidative stress and inflammation pathways occurring following a challenge with bacterial LPS.

## Methods

### Reagents

Arginine (Arg), Citrulline (Cit), Putrescine (Put), Spermidine (Spd), Spermine (Spm), methionine (Met), methionine sulfoxide (MetSO), S-adenosylmethionine (SAM), S-adenosylhomocysteine (SAH), homocysteine (Hcy), homocystine (Hcy2), cystathionine (Cysth), cysteine (Cys), cystine (Cys2), glutathione (GSH), glutathione-disulfide (GSSG), cysteinyl-glycine (Cysgly), cysteinyl-glycine disulfide (Cysgly2), glycine (Gly), glutamine (Gln), and glutamate (Glu), of highest analytical grade were purchased from Sigma (St. Louis, MO). Homoglutathione was purchased from BACHEM. Stable isotopes, methionine-methyl-D_3_, Homocystine (3,3,3’,3’,4,4,4’,4’-D_8_), Cystine (3,3,3’3’-D_4_), and Glycine (^13^C_2_, ^15^N) were purchased from Cambridge Isotopes. S-Adenosylmethionine (methyl-D_3_) was obtained from CDN Isotopes (Quebec, Canada). All HPLC solvents were of the analytical grade available from Fisher Scientific. The EZ-FAAST kit used for sample and standard derivatization was purchased from Phenomenex (Torrance, CA).

### Cell culture conditions

RAW267.4 mouse macrophage cells (RAW) in phenol-free Dulbecco’s minimal essential media (DMEM; Sigma) containing 5% heat-inactivated fetal bovine serum (FBS) were plated overnight in 6 well plates (2 × 10^6^ cells/well). To profile the concentration-dependent effects of LPS on amino acid metabolism, plated cells were incubated in media treated with or without 0.1, 1, 10, 100, and 1000 ng/ml bacterial lipopolysaccharide (LPS; type 055:B5) at 37°C for 24 hrs. For the time course experiments, cells were challenged with 10 ng/ml LPS at 37°C for 2, 4, or 6 hrs. To quantify amino acid metabolites, cells were detached from the plate with Trypsin and washed twice with pre-warmed phosphate buffered saline. Cell pellets were resuspended in 200 μl PBS buffer; cell number and viability were checked by tryphan blue exclusion assay. To quantify redox states of thiol amino acid metabolites, cell pellet suspensions were mixed with an equal volume of 20 mM iodoacetamide (IAM) dissolved in 0.1 M Tris-Cl buffer (pH 8.0) for 1 hr. Following IAM treatment, samples were deproteinized by mixing with an equal volume of 10% perchloric acid containing 0.2 mM DTPA and centrifuged at 13000 rpm for 5 min. Acidified supernatants were collected and stored at −80°C until analysis.

### Derivatization procedures

Stock solutions (10 mM) of amino acid standards were prepared in 5% perchloric acid containing 0.5 mM DTPA. An internal standards mix contained 10 μM solution of arginine (^15^N_2_), glycine (^13^C_2_, ^15^N), methionine (methyl-D_3_), S-adenosylmethionione (methyl-D_3_), cystine 3,3’3’3’-D_4_) and homocystine (3,3,3’3’4,4,4’4’-D_8_) stable isotopes. Standards were diluted to working concentrations of 0.05, 0.5, 5, and 50 μM in 5% PCA solution. Derivatization of acidified cell lysates and media was performed as previously described [37]. Briefly, 20 μl of internal standard solution was added to acidified cell lysates (180 μl). To these samples, 70 μl of KOH/tetrahydroborate buffer was added and centrifuged at 12,000 × g. Supernatants (200 μl) were derivatized using the EZ-FAAST Kit per manufacturer’s protocol.

### HPLC conditions

HPLC analysis was performed on the Shimadzu LC-10AD HPLC system equipped with the SIL-10 AVP auto-sampler, and two LC-AD10 pumps. The chromatographic separation was carried out on a 25 mm X 2 mm column supplied by EZ-FAAST (Phenomenex CA). The mobile phase was composed of methanol:water (80:20 v/v) containing 1 mM ammonium formate and the total flow rate was set at 0.25 ml/min.10 μl of each standards plus samples solution was injected with a total run time of 15 min.

### Mass spectrometer settings

Detection of derivatized metabolites and internal standards was carried out with Quattro LC mass spectrometer (Micromass; Waters, MA) in electrospray positive ionization mode. Results were quantified using the MassLynx 3.3 software. Capillary voltage was set to 3 kV, source temperature to 130°C, and nebulizer gas temperature to 400°C. The nebulizer and analysis cell gas flows (both nitrogen) were set at 80 and 800 L/h, respectively. The low and high mass resolutions were fixed at 15 for both the first and third quadrupole mass analyzers. The collision gas (argon) pressure in the second quadrupole was set at 1.5 mbar. The photomultiplier was fixed at 650. The dwell time and the interchannel delay were fixed at 0.2 and 0.01 s, respectively. The optimized cone voltages and collision energy settings for metabolites detected are listed in Additional file 1: Table S1.

### Statistical analysis

Statistical significance (p < 0.05) was determined using two-way ANOVA followed by a Dunnett’s post hoc tests for multiple comparisons between controls and cells treated with different concentrations of LPS and at different time points.

## Results

### LPS-dependent changes in the RAW macrophage amino acid profile

LPS activates macrophage via the classical activation pathway by initiating the Toll-like receptor 4 (TLR-4) and MD2-dependent redox sensitive inflammatory signaling cascade [38]. In LPS-stimulated macrophages, changes in gene expression have been detected as early as 2 hrs after challenge for early-response genes, whereas changes in late-response genes have not been detected until ~24 hrs after challenge [39].

As shown in Table 1, after 24 hrs incubation of RAW cells in the presence of 1 μg/ml LPS, cellular concentrations of 60% (18/34) of the metabolites measured were significantly altered compared to untreated cultures; concentrations of 11 increased, while 7 decreased. Metabolites that increased (Table 1) were primarily intermediates involved in either Arg or SAA metabolism (Figure 1). The two greatest increases were for Cit and Orn, intermediates produced by NOS and arginase enzymes respectively. Of these two metabolites, Cit exhibited the larger increase, with levels rising from 0.2 ± 0.02 nmol/mg protein in untreated cells to 10.3 ± 1.8 nmol/mg in LPS-treated cells (p < 0.0001). Orn, an arginase-mediated metabolite of arginine (Figure 1), increased to a lesser extent than Cit, approximately 7-fold (Table 1). Put, the rate-limiting substrate for cellular polyamines (Figure 1), also increased to a similar extent, approximately 5-fold (Table 1). To rule out the possibility of artifacts introduced by IAM incubation, intracellular concentrations of Arg, Cit and Orn were extracted with perchloric acid without IAM pretreatment with similar results (Additional file 2: Figure S1). This suggests that IAM did not cause incomplete arginase inactivation during the incubation period and it did not contribute to the LPS-dependent increase in intracellular Orn.

**Table 1:** LPS-induced changes in cellular amino acid metabolites

**Metabolite**	**Control**^**a**^**(nmol/mg protein)**	**LPS**^**b**^**(nmol/mg protein)**	**% CHANGE**^**c**^**from Control**	**Pathway**^**d**^	**Sig**^**e**^
**Cit**	0.2 ± 0.02	10.3 ± 1.8	5050.0	Arg Met	< 0.0001
**Orn**	0.7 ± 0.3	5.78 ± 0.8	752.9	Arg Met	< 0.0001
**Put**	1.2 ± 0.2	7.5 ± 2.4	525.0	Arg Met	< 0.0001
**Cys**	2.1 ± 0.7	6.2 ± 1.7	195.2	SAA Met	< 0.01
**Gln**	6.2 ± 0.5	16.1 ± 0.5	159.7	Arg Met	< 0.01
**Ile+Leu**	1.3 ± 0.2	3.3 ± 0.8	153.8	BCAA	< 0.05
**Phe**	0.6 ± 0.6	1.0 ± 0.3	66.7	EAA	< 0.05
**Hcy**	0.03 ± 0.01	0.05 ± 0.02	66.7	SAA Met	< 0.05
**Ser**	1.6 ± 0.2	2.2 ± 0.4	37.5	SAA Met	< 0.05
**Met**	4.0 ± 0.4	5.4 ± 0.3	35.0	SAA Met	< 0.05
**GSSG**	0.4 ± 0.1	0.5 ± 0.1	25.0	SAA Met	< 0.05
**Asn**	2.3 ± 0.3	2.6 ± 0.5	13.0		
**Pro**	5.4 ± 1.6	6.0 ± 1.3	11.1		
**Lys**	13.23 ± 2.85	14.61 ± 4.34	10.4		
**Ala**	3.3 ± 0.8	3.6 ± 0.34	9.1		
**His**	1.5 ± 0.5	1.6 ± 0.2	6.7		
**Spd**	13.3 ± 2.1	13.7 ± 2.3	3.0		
**Trp**	0.4 ± 0.1	0.4 ± 0.1	0.0		
**Tyr**	0.7 ± 0.3	0.7 ± 0.24	0.0		
**Val**	0.7 ± 0.3	0.7 ± 0.2	0.0		
**SAM**	0.2 ± 0.04	2.2 ± 0.2	−7.1		
**Thr**	9.2 ± 2.5	8.3 ± 1.9	−9.8		
**GSH**	46.8 ± 2.4	37 ± 4.9	−20.9	SAA Met	< 0.05
**Glu**	37.5 ± 3.8	24.9 ± 5.2	−33.6	SAA Met	< 0.05
**Gly**	25.4 ± 1.0	16.0 ± 1.1	−37.0	SAA Met	< 0.05
**SAH**	0.2 ± 0.03	0.1 ± 0.02	−50.0	SAA Met	< 0.01
**Spm**	23.8 ± 1.5	8.23 ± 6.2	−65.4	Arg Met	< 0.01
**Arg**	3.2 ± 0.13	1.1 ± 0.11	−65.6	Arg Met	< 0.0001
**Asp**	57.8 ± 13.8	15.8 ± 4.0	−72.7	Arg Met	< 0.0001

RAW macrophage cells (2 × 10^6^) were incubated in the absence (control) or presence of 1 μg/ml LPS (LPS) for 24 hrs. Cellular metabolites were extracted and analyzed as described in Methods. **a,b -** Metabolites quantified and their corresponding concentrations are listed. Values presented are averages from 5 experiments (mean ± SD). **c-** % change for each of the metabolites in LPS treated cells from control cultures are listed. **d-** Metabolic pathways for metabolites that were significantly altered by LPS are listed. **e-** * denotes statistical significance of the differences detected.

Abbreviations: Alanine – Ala; Arginine – Arg; Arginine metabolism - Arg Met; Asparagine – Asn; Aspartate – Asp; Branched Chain Amino Acids - BCAA Citrulline – Cit; Cysteine – Cys; Essential Amino Acids - EAA Glutamate – Glu; Glutamine – Gln; Glutathione – GSH; Glutathione disulfide – GSSG; Glycine – Gly; Histidine – His; Homocysteine – Hcy; Isoleucine +Leucine – Ile-Leu; Lysine – Lys; Methionine – Met; Ornithine – Orn; Proline - Pro; Putrescine – Put; Phenylalanine – Phe; Sulfur Amino Acid Metabolism - SAA Met; S-adenosylmethionine – SAM; S-adenosylhomocysteine – SAH; Serine – Ser; Spermidine – Spd; Spermine – Spm; Threonine – Thr; Tryptophan – Trp; Tyrosine – Tyr; Valine – Val.

Though their response was modest in comparison to Arg-related intermediates, certain SAA metabolites were also responsive to LPS treatment. Most notably, Cys, the rate-limiting precursor of cellular GSH, increased several-fold in LPS treated cells, from 2.1 ± 0.7 to 6.2 ± 1.7 nmol/mg protein. Gln is metabolized to form Glu, which serves as a precurser to Arg and GSH. Gln concentration was increased by ~60% in LPS-treated compared to untreated cells (Table 1). Met and Hcy also increased by ~35 and 65%, respectively, following LPS treatment. These metabolites are key components of the transmethylation pathway, which supplies Cys required for GSH synthesis. Lastly, cellular concentrations of GSSG, the homodisulfide of GSH, increased by ~25% in LPS treated cells.

In addition to the 6 metabolites that increased by more than 2-fold following LPS exposure, 7 compounds were significantly decreased. Intracellular Arg and Asp significantly decreased by 65.6 and 72.7% respectively. Asp is a substrate required for Arg regeneration via the argininosuccinate lyase pathway [29,40]. The decreases in both Arg and Asp suggest that LPS imposes an acute depletion of cellular Arg equivalents. Despite a significant increase in cellular Put, cellular levels of polyamines derived from Put decreased. SPM declined from 23.2 ± 1.5 nmol/mg protein in control cultures to 8.2 nmol/mg protein in LPS treated cells. Cellular SAH, Gly, Glu and GSH showed more modest declines following LPS treatment. Compared to controls, LPS-treated cells contained ~50% less cellular SAH, a by-product of SAM methylation reactions. Concentrations of Glu and Gly, constituents of cellular GSH, both declined by ~30%. Cellular GSH decreased from 46.6 ± 2.4 in untreated cells to 37 ± 4.9 nmol/mg protein in LPS-treated cells. This ~20% loss in cellular GSH, along with similar increases in GSSG concentration, indicate that LPS treatment shifted cellular thiol redox to a more oxidized state.

### LPS concentration-dependent effects on Arg metabolism

**Figure 2:**
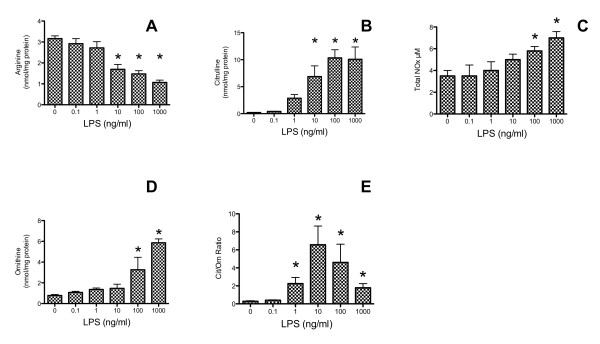
**NO formation in LPS-activated macrophage is regulated by changes in iNOS and Arginase activities.** The concentration dependent effects of LPS on intracellular Arg (panel **A**) and its metabolic products, Cit (panel **B**) and Orn (panel **D**) are shown. The Cit/Orn ratio, which indicates the balance between NOS and arginase dependent pathway is plotted in (panel **E**) and compared to the media total media nitrate/nitrite (NOx; panel **C**) concentration. Raw cells (2 × 10^6^ cells) were either left untreated or incubated with 0.1, 1,10, 100 and 1000 ng/ml LPS at 37°C for 24 hrs.

Evidence suggests that the metabolic fate of Arg is a critical determinant of classical or alternative activation pathways in macrophage cells [9-11]. Effects on the steady-state concentrations of Arg and two major Arg-derived metabolites, Cit and Orn, were measured 24 hrs after treatment with 0, 0.1, 1, 10, 100, and 1,000 ng/ml LPS (Figure 2). As shown, significant declines in Arg and increases in Cit were observed when LPS concentrations were at 10 ng/ml and above. Orn also increased significantly, but only at the two highest LPS concentrations.

The Cit/Orn ratio increased significantly at lower concentrations of LPS (1 ng/ml) than required to cause a significant increase in either Cit or Orn individually. The maximal increase in the Cit/Orn ratio was observed when cells were treated with 10 ng/ml LPS. At higher concentrations of LPS (100 and 1000 ng/ml), the cellular Cit/Orn ratio decreased to 4.5 and 1.8 from the maximal ratio of 6.6 obtained at 10 ng/ml LPS. The data indicate that the increase in Orn, which occurred at concentrations of LPS at or above 100 ng/ml, signals a shift in Arg consumption toward urea generation. Orn is a precursor to both proline and the polyamines spermidine and spermine. The concentrations of Pro, and Spd remained unchanged across all concentrations of LPS tested (Table 2). Thus, while Orn increased at higher LPS concentrations, this did not result in increased generation of its products.

**Table 2:** LPS-dependent changes in ornithine derived products

				**LPS Concentrations**		
**Metabolite**	**Control**	**0.1 ng/ml**	**1 ng/ml**	**10 ng/ml**	**100 ng/ml**	**1000 ng/ml**
**Orn**	0.78 ± 0.20	1.07 ± 0.25	1.36 ± 0.31	1.47 ± 0.88	3.26 ± 0.24^*^	5.86 ± 0.83^**^
**Put**	1.21 ± 0.39	2.88 ± 0.98	6.05 ± 2.8^**^	7.94 ± 3.11^**^	9.56 ± 4.28^**^	9.31 ± 3.42^**^
**Pro**	5.45 ± 1.62	6.77 ± 1.36	7.81 ± 2.90	7.38 ± 1.98	7.07 ± 1.82	6.04 ± 1.33
**Spd**	13.32 ± 2.09	15.72 ± 2.28	13.67 ± 2.35	13.93 ± 2.09	13.66 ± 1.60	13.74 ± 2.38
**Spm**	23.82 ± 1.55	14.07 ± 3.05^*^	12.60 ± 2.48^*^	10.69 ± 2.75^**^	7.39 ± 1.86^**^	6.75 ± 2.04^**^

RAW cells (2 × 10^6^) cells were incubated in the absence (control) or in the presence of 0.1, 1, 10, 100 and 1000 ng/ml LPS (LPS) for 24 hrs. Cellular metabolites were extracted and analyzed as described in methods. Results are an average of 3 independent experiments. * and ** denotes *p < 0.05 or p < 0.01, respectively.*

### Threshold concentrations of LPS

As shown in Table 1, LPS treatment for 24 hrs strongly increased Arg metabolism. Though less prominent than changes in the Arg pathway, SAA metabolites (notably GSH, which indicates the redox state of the cell) decreased following LPS treatment. These results suggest that Arg and SAA metabolites are highly responsive to LPS-dependent classical M1 activation and may be useful as surrogate markers of inflammation.

The Arg and SAA metabolites that exhibited the greatest change on LPS treatment were Arg, Asp, Orn, and Cit. Arg and Asp exhibited the greatest decrease after LPS treatment, while their products Orn and Cit exhibited the greatest increase. We hypothesized that a ratio of the sums of these metabolites: (Cit + Orn)/(Arg + Asp), which we term the CO/AA ratio, would be highly sensitive to metabolic shifts following LPS treatment. The relative sensitivity of this ratio to LPS was compared to several established amino acid ratios used as biomarkers of LPS- dependent inflammation: SAM/SAH [40], GSH/GSSG [41,42], Spd/Spm [43], and Gln/Glu [44]. In addition to these markers, the media concentration of NOx, was also measured because it is a metabolite-independent measure of RAW cell activication and inflammation [45].

**Figure 3:**
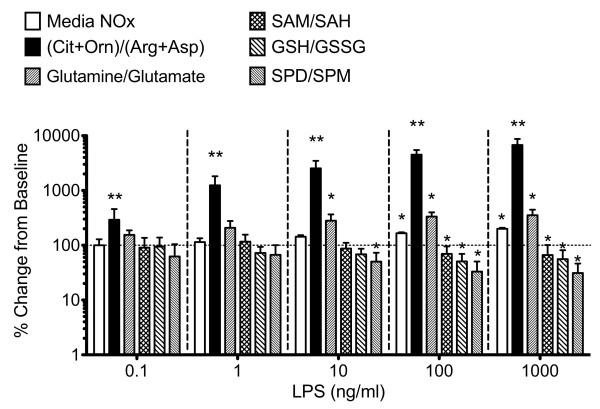
**The cellular CO/AA ratio is the most sensitive amino acid-based biomarker of LPS activation.** The relative sensitivity of the CO/AA ratio to varying concentrations of LPS was compared to total media nitrate/nitrite (NOx), Gln/Glu, SAM/SAH, GSH/GSSG, AND Spd/Spm ratios. Raw cells (2 × 10^6^ cells) were either left untreated or incubated with 0.1, 1,10, 100 and 1000 ng/ml LPS at 37°C for 24 hrs. Media NOx concentrations and metabolite ratios obtained were normalized to baseline levels in untreated cells and the % changes from baseline were plotted (mean ± SD; N = 6). The CO/AA ratio was the sole indicator that was significantly changed, even at 0.1 ng/ml LPS, and further increased at higher LPS concentrations in a dose-dependent manner. At all concentrations of LPS tested, the changes in CO/AA ratio were greater than other metabolic indices of macrophage activation. * denotes p < 0.05; ** denotes p < 0.001.

As shown in Figure 3, significant increases in total media NOx following 24 hrs incubation with LPS were detectable only when LPS concentrations were ≥ 100 ng/ml. The total media NOx concentration increased from baseline values of 3.5 ± 0.5 μM to 5.8 ± 0.4 and 7.0 ± 0.6 μM in cells treated with 100 and 1000 ng/ml LPS, respectively. In contrast, 0.1 ng/ml LPS was sufficient to cause a statistically significant 290 ± 166% increase in the CO/AA ratio (p < 0.01). Further increasing LPS concentrations to 1, 10 and 100 and 1000 ng/ml caused the intracellular CO/AA ratio to rise by 1241 ± 576, 2530 ± 945, 4497 ± 979, and 6775 ± 1969%, respectively.

As evident in Figure 3, the CO/AA ratio was most sensitive to metabolic shifts following LPS treatment at all concentrations tested. Significant alterations in other amino acid metabolite markers were only observable after treatment with at least 10 ng/ml LPS. At 10 ng/ml LPS the Gln/Glu ratio increased by 280 ± 69%, whereas the Spd/Spm ratio decreased by approximately 50%. As shown, LPS-dependent changes in metabolite ratios linked to cellular sulfur amino acid metabolism, such as SAM/SAH and GSH/GSSG were only observed at LPS concentrations ≥100 ng/ml.

### Earliest detectable metabolic change following LPS treatment

**Figure 4:**
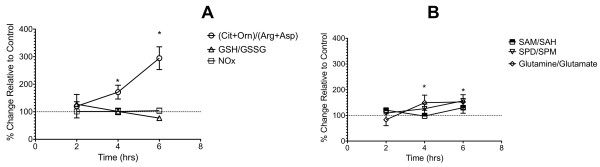
**Shifts in the macrophage CO/AA ratio temporally precede changes in other amino acid metabolites and total media NOx levels.** Temporal changes in the CO/AA ratio were plotted against other metabolic indices of macrophage activation. Briefly, RAW cells (2 × 10^6^ cells) were either left untreated or treated with 10 ng/ml LPS for 2, 4 or 6 hrs. At each time point, cells were harvested and metabolites (mean ± SD; N = 6) were quantified as described in Methods. Panel A shows time-dependent changes in the ratios of CO/AA and GSH/GSSG, and total media nitrate/nitrite concentrations (NOx). * denotes statistical significance ( P <0.05).

To ascertain the earliest time-point following LPS treatment at which metabolic change was detected by the CO/AA ratio as compared to the other biomarkers discussed above, RAW cells were either left untreated or treated with 10 ng/ml LPS for 2, 4, or 6 hrs. As shown in Figure 4A, at this concentration of LPS, the CO/AA ratio significantly increased by 171 ± 25% (p < 0.01) after 4 hrs following LPS treatment, and increased by 294 ± 42% at 6 hrs. In contrast, total media NOx concentrations and cellular GSH/GSSG ratios remained unchanged relative to controls. Glutamine/glutamate and Spd/Spm ratios significantly increased 4 hrs following LPS challenge (Figure 4B), however the SAM/SAH ratio, which measures the cellular methylation capacity, did not change during the first 6 hrs following LPS addition (Figure 4B).

In summary, the CO/AA ratio changed significantly at lower LPS concentrations and at earlier times following challenge than the other metabolic indices of macrophage activation to which it was compared.

## Discussion

In the present work, a comprehensive Arg/thiol redox amino acid metabolomics assay was used to monitor amino acid metabolic changes following LPS challenge of RAW macrophage cells. The Arg-Cit-NO cycle and arginase pathways were identified as most sensitive to LPS. This assay was also useful in determining the temporal relastionships between these pathways and cellular redox regulation.

As shown in Table 1 and Figures 3– 4, Arg, Asp, Cit and Orn were most significantly affected by LPS treatment. Since these metabolites are involved in Arg catabolism and synthesis, these results indicate that Arg metabolic pathways are most immediately impacted by LPS. The concentration ratio (Cit+Orn)/(Arg+Asp) was most sensitive to LPS challenge, changing significantly at lower concentrations and earlier times following LPS treatment compared to total media NOx and other commonly used metabolic biomarkers of cellular methylation, redox, polyamine and nitrogen balance status (Figure 3).

Because the CO/AA ratio significantly changed prior to cellular GSH/GSSG redox change, it appears to be a redox independent biomarker of macrophage activation. Thus, simultaneous quantification of CO/AA and thiol redox ratios allows for concurrent analysis of metabolic shifts during cellular inflammation and oxidative stress.

### Arginase and iNOS regulation

Results presented here reveal the importance of arginase in iNOS regulation. LPS is known to acutely stimulate iNOS within the first 24 hrs of macrophage activation [11,46] and the induction of iNOS and subsequent NO generation are critical to macrophage innate immune functions [47]. In contrast, the role of arginase in iNOS regulation during the initial phases of macrophage activation is less well understood. The ratio of Cit/Orn increased linearly with LPS concentration, with maximal effects observed at 10 ng/L, declining at 100 and 1000 ng/ml, due to the rise in Orn (Figure 2). These data suggest that the relatively low sensitivity of arginase enzymes to LPS at low concentrations allows for maximal rates of iNOS activity, but at higher LPS concentrations, induction of arginase resulting in increased Orn, may prevent the toxicity of excess NO generation. Arginase inhibition therapy has been proposed to improve endothelial function by increasing Arg available for NO generation [48,49]. Results reported here suggest that arginase activity differs depending on LPS burden, and that arginase inhibition when LPS is high may exacerbate inflammation by excessive NO production.

### Type I and Type II arginase isoforms

Results presented here suggest that Arg-derived metabolites may be useful in determining the type of arginase isoform expressed in cells [11]. Macrophage express both type I and type II arginases. The type I (cytoplasmic) isoform is typically associated with the TH2 alternative macrophage activation pathway, which is characterized by elevated polyamine and proline production [9,49,50]. In contrast, the type II (mitochondrial) form of arginase is induced only by TH1 classical activation, and is charcterized by the lack of polyamine and proline production. LPS induces TH1 classical activation and thus only type II arginase is induced [11]. Results presented here are consistent with TH1 activation since this increase was not accompanied by an increase in spermidine, spermine, or proline (Table 2) , though Orn was increased at very high LPS concentrations ( > 100 ng/ml). Thus, increased Orn/polyamine or proline ratios may be indicative of arginase II induction whereas the opposite may indicate arginase I induction. This suggestion is plausible because the downstream enzymes for polyamine and proline synthesis are cytosolic enzymes and Orn synthesized in mitochondria may not feed into these pathways.

### A role for Asp in Arg synthesis in macrophage?

LPS-stimulated macrophage metabolize Arg at a greatly enhanced rate, and therefore a continous supply of Arg is needed by these cells to support their immune functions. To meet this demand, LPS-activated macrophage cells increase both the uptake and synthesis of Arg [29,51,52]. In response to LPS (1 μg/ml) treatment (Table 1 and Figure 2), the concentrations of Cit and Orn, the two metabolic products of Arg, increased significantly and greatly exceeded the amount of Arg loss from levels found in untreated cells. As this was a steady-state measurement, the extent of Arg loss in LPS activated cells may underestimate the actual rate of Arg uptake and resynthesis.

The cellular uptake of Arg is mediated by the cationic amino acid transporter 2 (CAT2) [53]. CAT2 is coinduced with iNOS in LPS-treated macrophages [53]. Inhibition of CAT2 decreases iNOS-dependent Cit/NO generation without impacting the level of iNOS protein abundance [53]. Although Arg is its preferred substrate, CAT2 is also capable of transporting other cationic amino acids, such as Orn and Lys, that can potentially compete with Arg import and reduce Cit/NO output. However, this is unlikey to be the case in this model because the concentration of Lys or Orn in the media was far below the concentration required for competitive inhibition of Arg uptake and subsequent NO and/or Cit synthesis [51,53].

Cit (produced along with NO from Arg) can synthesize Arg by the combined actions of the argininosuccinate synthetase (ASS) and argininosuccinate lyase (ASL) [28]. ASS and ASL are known to increase in RAW macrophage following LPS challenge [28,29,54]. Though Asp is required for Cit-dependent Arg synthesis by the ASS/ASL pathway, a role for Asp in Arg synthesis in macrophage has not been established. Although this report does not conclusively establish the metabolic fate of Asp on LPS challenge, the large decrease in both Asp and Arg (Table 1) suggest potential involvement of Asp in Arg synthesis. The contribution of Asp to support Arg-Cit-NO cycle may be particularly more important in RAW macrophages because these cells may not contain active carbamyl phosphate synthetase (CPS) or ornithine transcarbamylase enzymes (OTC) required for converting other amino acids, such as Pro, to synthesize Cit and Arg [29].

It should be noted that unlike plasma or other widely used media such as RPMI, the DMEM media used in this study lacks Ala, Pro, Glu, Asp and Asn. It is possible that this deficiency would have prevented the utilization of these amino acids to regenerate Arg, that would otherwise occur under amino acid replete conditions. However, cellular biosynthesis of these non-essential amino acids would also be expected, which would be expected to mitigate any deficiency. While deficiency could theoretical impact the rate of Arg replenishment, this is unlikely to alter our major findings because availability of these substrates do not directly modulate LPS-dependent up-regulation of iNOS and arginase expression.

### Oxidation of cellular GSH following LPS challenge occurs secondary to iNOS activation

Oxidative stress modulates the LPS-dependent activation of pro-inflammatory genes, including the iNOS protein [55-57]. Nuclear Factor-kappa-light-chain-enhancer of activated B cells (NFkB) is a key inflammatory transcription factor responsible for up-regulating inflammatory genes following LPS exposure [56]. LPS increases macrophage oxidant generation from multiple sources such as mitochondria [58] and the NADPH oxidase enzyme [59]. ROS mediated oxidation of critical thiol in MAP kinase phosphatase-1, a key phosphatase enzyme in macrophage cells, has been shown to be involved in the activation of upstream MAP kinase proteins (p38, JNK, and Erk1/2) responsible for NFkB activation [46]. Because of the potential role of oxidative stress in iNOS activation, the oxidation of the GSH pool may precede LPS-dependent NFkB activation. However, the analysis here (Figure 4A) indicates that oxidation of cellular GSH, following LPS challenge, occurs secondary to increased Arg-Cit-NO pathway activity, as no significant declines in GSH/GSSG ratios are observed during the first 6 hrs following LPS addition. This result suggests that the initial activation of iNOS does not require a GSH redox pool oxidation. However, it is possible that subsequent oxidation of the GSH pool may play a role in propagation rather than the initiation of the LPS inflammation signaling.

### A new mechanism contributing to inflammatory signaling?

In addition to GSH, LPS addition caused the elevation in cellular Cys and Hcy. Cys and Hcy exert pro-oxidant effects in cells by oxidizing protein thiols through S-cysteinylation or S-homocysteinylation [12,16,60,61]. However, there is no evidence that Cys and Hcy have any involvement in inflammatory signaling. The rise in Cys and Hcy observed after LPS challenge (Table 1), can cause oxidation of protein disulfides by means of mixed disulfide formation. The potential role of Cys or Hcy-dependent S-thiolation in macrophage redox signaling is currently unknown. Given the reactivity of Cys and Hcy to protein S-thiolation, the LPS-dependent increase in their levels, as seen in this study, indicates that they may represent non-oxidant mediated redox signaling mechanism.

### Clinical implications

LPS has been implicated not only in sepsis but also as a potential trigger of subclinical inflammation in obesity and in cardiovascular diseases [62,63]. A possible mechanism for inflammation in these diseases may involve activation of macrophage cells by low threshold grade LPS (1 ng/ml), which cooperatively enhances the pro-atherogenic effects of oxidized LDL [63]. Detection of cellular effects of such low levels of LPS is challenging and the metabolomics data presented indicate that the OC/AA ratio is significantly increased in response to low concentration of LPS (0.1 ng/ml) than existing biomarkers (Figure 3). When combined with its ability to monitor major physiological small molecule thiol redox states, this assay may be useful in quantifying the onset of sub-clinical inflammation mediated by monocytes/macrophage cells.

Prospective human studies have shown that plasma ratios of Arg/Cit + Orn is associated with increased risk for developing adverse cardiovascular disease outcomes in healthy adults and risk of morbidity and mortality in sickle cell patients [64,65]. Plasma thiol redox perturbations occur in the course of aging and development of age-related diseases [12,16]. Inflammation in healthy individuals with or without oxidative stress may have different clinical consequences. The ability to measure both processes, as in this present study, may be clinically useful in further clarifying the role of inflammation in disease. During its development phase, this assay was succesfully applied to monitor leukocyte oxidative stress in children with austism spectrum disease [66], erythrocyte oxidative stress in sickle cell patients [67], plasma and erythryocyte oxidative stress in thalassemia patients [37], oxidative stress in lung fibroblasts caused by cystic fibrosis conductance transporter mutation [68] and cigarette exposure [69]. Inflammation in healthy individuals with or without oxidative stress may have different clinical consequences. The ability to quantify shifts in both inflammation and redox pathways, as presented in this paper, may be clinically useful in further clarifying the role of inflammation in diseases.

## Conclusions

Amino acid metabolism changes with activation states of macrophage. However, few studies have explored the potential of amino acid metabolites as sensitive biomarkers for inflammation and redox stress. By comprehensively quantifying Arg and SAAs and their metabolic intermediates, the early metabolic shifts during macrophage activation were established, and the CO/AA ratio was identified as a new sensitive biomarker of macrophage inflammation. Additionally, mechanistic insights into how specific changes in Arg-Cit-NO Cycle relate to cellular redox and polyamine balance were also suggested. Specifically, data suggest that GSH redox loss is not required for iNOS activation. The concentration-dependent effects of LPS on Cit/Orn balance and polyamines suggests that activation of mitochondrial type II arginase is required for preventing excessive iNOS activity, but does not result in increased cellular polyamine content. It is suggested that monitoring macrophage health in human plasma may permit early identification of sub-clinical inflammation and oxidative stress.

## Abbreviations

Ala: Alanine; Arg: Arginine; Asn: Asparagine; Asp: Aspartate; ASL: Argninosuccinate Lyase; ASS: Argininosuccinate Synthetase; BHMT: Betaine Hydroxymethyl Transferase; Cit: Citrulline; Cys: Cysteine; CBS: Cystathionine Beta-synthase; CL: Cystathionine gamma-Lyase; CPS: carbamyl phosphate synthetase; GGT: Gamma-Glutamyl Transpeptidase; GCL: Gamma-Glutamylcysteine Ligase; GSH: Glutathione; GSSG: Glutathione disulfide; GS: Glutathione synthase; Glu: Glutamate; Gln: Glutamine; Gln Syn: Glutamine Synthase; Gly: Glycine; His: Histidine; Hcy: Homocysteine; IAM: Iodoacetamide; Ile-Leu: Isoleucine +Leucine; IPCF: Isopropylchloroformate; LPS: Lipopolysaccharides; Met: Methionine; MAT: Methionine Adenosyl Transferase; MT: Methyl Transferase; MS: Methionine Synthase; NOS: Nitric Oxide Synthase; Orn: Ornithine; ODC: Ornithine Decarboxylase; OTC: Ornithine transcarbamylase; Pro: Proline; Put: Putrescine; PAO: Polyamine Oxidase; SAM: S-adenosylmethionine; SAMDC: S-Adenosylmehtionine Decarboxylase; SAH: S-adenosylhomocysteine; SAHH: S-adenosylhomocysteine hydrolyase; Ser: Serine; SPD: Spermidine; Spd Syn: Spermidine Synthase; Spm Syn: Spermine synthase; Spm: Spermine; SSAT: Spermidine/Spermine N1 acetyltransferase, Thr, Threonine; Trp: Tryptophan; Tyr: Tyrosine; Val: Valine.

## Competing interests

No conflicting interests.

## Authors' contributions

Kim, R and Lee, D performed the experiments. Suh, JH designed the study, performed mass spectrometry analysis, interpreted data, and wrote the manuscript. All authors read and approved the final manuscript.

## Supplementary Material

Additional file 1 **Table S1.** Mass spectrometric setting used in this study. Click here for file

Additional file 2 **Figure S1.** Effects of iodoacetamide (IAM) pretreatment on cellular arginine (Arg), citrulline (Cit) and ornithine (Orn) concentrations. The effects of iodoacetamide pretreatment on intracellular Arg (Panel A), Cit (Panel B) and Orn (Panel C) concentration are shown. RAW cells (2 × 10^6^) were left untreated (control) or treated with 0.1, 1, 10, 100 and 1000 ng/ml LPS (LPS) for 24 hrs at 37°C. For extraction without IAM, cells were harvested and washed with phosphate buffered saline and immediatedly treated with perchloric acid. For extraction with IAM, cells were pre-incubated with IAM solution for 1 hr prior to perchloric acid treatment. Metabolite concentrations were normalized to protein concentrations. Results show no significant differences between cells treated with or without IAM. Click here for file
