# Supplementary material for: A new metabolomic assay to examine inflammation and redox pathways following LPS challenge
Source: J Inflamm (Lond). 2012 Oct 4;9:37. doi: 10.1186/1476-9255-9-37 (PMC3507808; doi:10.1186/1476-9255-9-37)
Supplement: Additional file 1 — Table S1. Mass spectrometric setting used in this study. [file 1476-9255-9-37-S1.doc]

| Supplementary Table 1. Mean retention times and analyte-specific MS parameters | | | | |  |  |
| --- | --- | --- | --- | --- | --- | --- |
| IAM + IPCF Derivative | | | | | | |
| Analyte | MW | Precursor Ion [M+H]^+^ | Product Ion | Retention Time | Cone Voltage | Collision Energy |
| Homocysteine | 135.2 | 321 | 142 | 4.1 | 28 | 15 |
| Cysteine | 121.2 | 307 | 116 | 3.9 | 28 | 18 |
| Glutathione | 307.3 | 535 | 418 | 4.7 | 28 | 18 |
| Cysteinylglycine | 178.2 | 364 | 116 | 3.5 | 28 | 20 |
| Homoglutathione | 321.3 | 549 | 418 | 5.2 | 28 | 18 |
| IPCF Derivative | | | | | | |
| Analyte | MW | Precursor Ion [M+H]^+^ | Product Ion | Retention Time | Cone Voltage | Collision Energy |
| 1-Methyl histidine | 169.2 | 298 | 96 | 4.3 | 30 | 18 |
| 3-Methyl histidine | 169.2 | 298 | 210 | 4.4 | 30 | 18 |
| 4-hydroxylproline | 131.0 | 260 | 200 | 4.8 | 28 | 15 |
| Alanine | 89.1 | 218 | 130 | 5.8 | 20 | 15 |
| alpha-aminoadipic acid | 161.2 | 332 | 244 | 10.7 | 25 | 15 |
| Arginine | 174.2 | 303 | 286 | 3.1 | 35 | 18 |
| Arginine N_15_ | 213.0 | 305 | 287 | 3.1 | 35 | 18 |
| Asparagine | 132.1 | 243 | 115 | 4.2 | 25 | 15 |
| Aspartate | 133.1 | 304 | 216 | 9.4 (p), 8.9 (r) | 25 | 15 |
| beta-Alanine | 89.1 | 218 | 96 | 5.7 | 20 | 15 |
| Citrulline | 175.2 | 304 | 287 | 3.8 | 35 | 18 |
| Cystathionine | 222.3 | 479 | 230 | 12.5 | 35 | 16 |
| Cysteinylglycine disulfide | 297.4 | 611.5 | 363 | 9.1 | 35 | 20 |
| Cystine | 240.3 | 497.5 | 248 | 12.9 | 37 | 17 |
| Cystine-d_4_ | 244.3 | 501.5 | 250 | 12.9 | 37 | 17 |
| Ergothioneine | 229.3 | 402 | 358 | 4.1 | 25 | 15 |
| Glutamate | 147.1 | 318 | 258 | 9.5 | 20 | 12 |
| Glutamine | 146.1 | 275 | 172 | 3.7 | 18 | 12 |
| Glutathione disulfide | 612.6 | 953 | 836 | 14.2 | 58 | 20 |
| Glycine | 75.1 | 204 | 144 | 4.6 | 25 | 8 |
| Glycine C_13_, N_15_ | 78.1 | 207 | 147 | 4.6 | 25 | 8 |
| Histidine | 155.2 | 370 | 196 | 8.9 | 25 | 15 |
| Homocystine | 268.4 | 525.5 | 262 | 15.0 | 35 | 15 |
| Homocystine-d_8_ | 276.4 | 533 | 266 | 14.9 | 35 | 15 |
| Isoleucine | 131.2 | 260 | 172 | 11.5 | 20 | 15 |
| Leucine | 131.2 | 260 | 172 | 11.1 | 20 | 15 |
| Lysine | 146.2 | 361 | 301 | 8.9 | 25 | 12 |
| Methionine | 149.2 | 278 | 190 | 8.0 | 20 | 10 |
| Methionine D_3_ | 152.2 | 281 | 193 | 7.9 | 20 | 10 |
| Methionine Sulfoxide | 165.2 | 294 | 234 | 3.8 | 33 | 10 |
| Methionine Sulfoxide D_3_ | 168.2 | 297 | 237 | 3.8 | 33 | 10 |
| Ophthalmic acid | 289.3 | 460 | 343 | 6.6 | 20 | 8 |
| Ornithine | 168.6 | 347 | 287 | 7.7 | 30 | 12 |
| Phenylalanine | 165.2 | 294 | 206 | 11.2 | 30 | 12 |
| Proline | 115.1 | 244 | 156 | 8.1 | 20 | 12 |
| S-adenosylhomocysteine | 384.4 | 685 | 230 | 14.9 | 35 | 25 |
| S-adenosylmethionine | 398.4 | 699 | 230 | 6.3 | 35 | 18 |
| S-adenosylmethionine D_3_ | 401.4 | 702 | 230 | 6.3 | 35 | 18 |
| Sarcosine | 89.1 | 218 | 116 | 6.6 | 20 | 15 |
| Serine | 105.1 | 234 | 146 | 4.2 | 20 | 12 |
| Spermidine | 145.3 | 318 | 102 | 3.2 | 40 | 30 |
| Spermine | 202.3 | 461 | 144 | 4.6 | 45 | 25 |
| Thiaproline | 133.2 | 262 | 174 | 11.1 | 20 | 12 |
| Threonine | 119.1 | 248 | 160 | 4.8 | 18 | 12 |
| Tryptophan | 204.2 | 333 | 245 | 9.9 | 15 | 18 |
| Tyrosine | 181.2 | 396 | 308 | 13.6 | 25 | 15 |
| Tyrosine D_2_ | 183.2 | 398 | 310 | 13.6 | 25 | 15 |
| Valine | 117.2 | 246 | 158 | 9.3 | 20 | 12 |
