# Supplementary figures and images for: A new metabolomic assay to examine inflammation and redox pathways following LPS challenge
Source: J Inflamm (Lond). 2012 Oct 4;9:37. doi: 10.1186/1476-9255-9-37 (PMC3507808; doi:10.1186/1476-9255-9-37)

**A**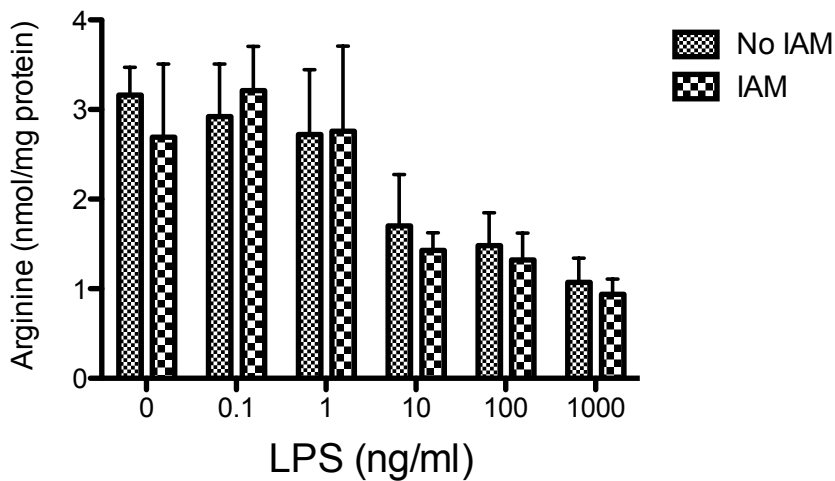**B**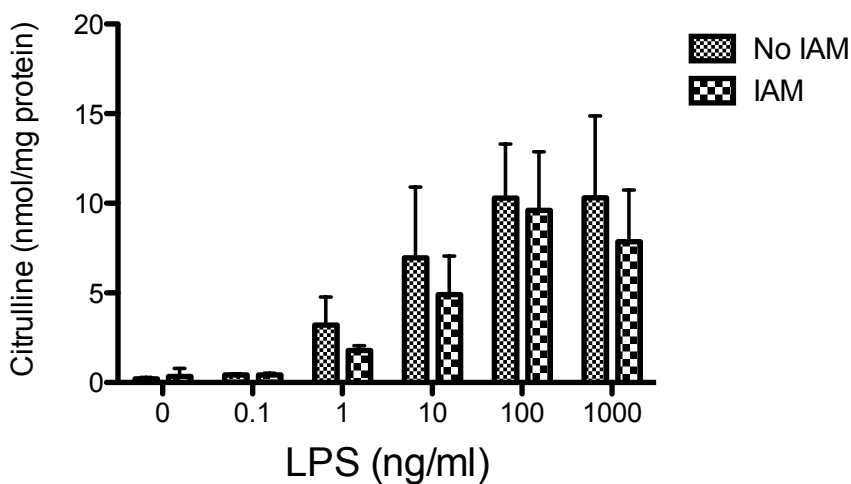**C**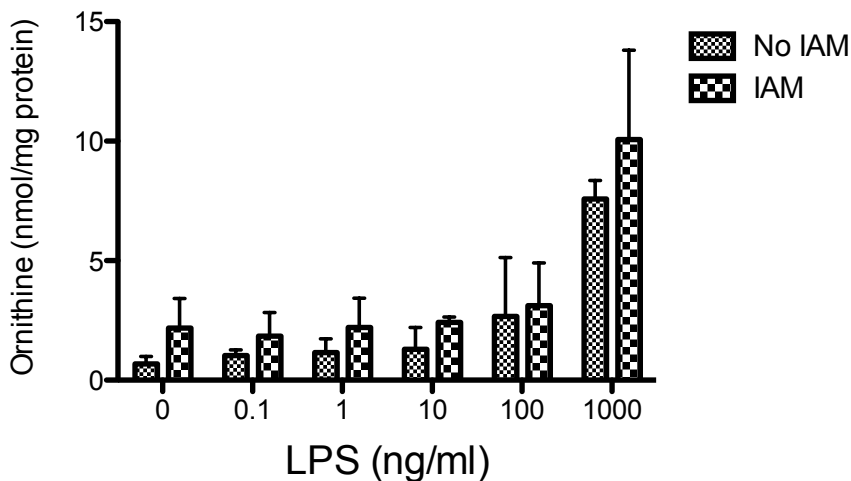

Supplement: Additional file 2 — Figure S1. Effects of iodoacetamide (IAM) pretreatment on cellular arginine (Arg), citrulline (Cit) and ornithine (Orn) concentrations. The effects of iodoacetamide pretreatment on intracellular Arg (Panel A), Cit (Panel B) and Orn (Panel C) concentration are shown. RAW cells (2 × 10^6^) were left untreated (control) or treated with 0.1, 1, 10, 100 and 1000 ng/ml LPS (LPS) for 24 hrs at 37°C. For extraction without IAM, cells were harvested and washed with phosphate buffered saline and immediatedly treated with perchloric acid. For extraction with IAM, cells were pre-incubated with IAM solution for 1 hr prior to perchloric acid treatment. Metabolite concentrations were normalized to protein concentrations. Results show no significant differences between cells treated with or without IAM. [file 1476-9255-9-37-S2.pdf]
